# Effects of far-off and close-up transition cow feeding on uterine health, postpartum anestrous interval, and reproductive outcomes in pasture-based dairy cows

**DOI:** 10.1186/s40104-019-0416-8

**Published:** 2020-02-06

**Authors:** S. Meier, J. K. Kay, B. Kuhn-Sherlock, A. Heiser, M. D. Mitchell, M. A. Crookenden, M. Vailati Riboni, J. J. Loor, J. R. Roche

**Affiliations:** 1 DairyNZ Limited, Private Bag 3221, Hamilton, 3240 New Zealand; 2 AgResearch, Hopkirk Research Institute, Grasslands Research Centre, Palmerston North, 4442 New Zealand; 3 Centre for Children’s Health Research, Queensland University of Technology, Level 6, 62 Graham Street, South Brisbane, Queensland 4101 Australia; 4 DairyNZ Limited, c/o University of Auckland, 3A Symonds St, Auckland, 1010 New Zealand; 5 Department of Animal Sciences, University of Illinois, Urbana, 61801 USA; 6 Ministry for Primary Industries-Manatū Ahu Matua, Charles Ferguson Tower, Pipitea, Wellington, 6140 New Zealand; 7 School of Biological Sciences, University of Auckland, Private Bag 92019, Auckland, 1142 New Zealand

**Keywords:** Close-up, Dairy cows, Far-off, Reproduction, Transition period

## Abstract

**Background:**

In seasonal, pasture-based, dairy production systems, cows must recover from calving and become pregnant within two to 3 months. To achieve this, the uterus must involute and ovulation must occur and continue at regular intervals. As these processes are affected by the cows’ nutritional or metabolic status post-calving, the objective of this study was to evaluate the effect of cow feeding strategies on uterine health, the length of postpartum anestrous interval, and reproductive outcomes. The treatments consisted of two feeding strategies during late-lactation and early dry period (far-off period; starting 4-month pre-calving) and three close-up dry period feeding regimes (1-month pre-calving) in a 2 × 3 factorial arrangement. We randomly assigned 150 cows to one of two far-off treatments. During late lactation, the herds (*n* = 75 cows per herd) were either control-fed (Controlfed) or over-fed (Overfed) to achieve a low or high body condition score (4-month pre-calving; BCS; ~ 4.25 and ~ 4.75; 10-point scale) at cessation of lactation. Within each of these treatments, three feeding levels were applied during the close-up period (1-month pre-calving): ~ 65% (Feed65), ~ 90% (Feed90), or ~ 120% (Feed120) of metabolizable energy (ME) intakes relative to pre-calving requirements.

**Results:**

Uterine health improved (i.e. polymorphonucleated (PMN) cells declined) with increased feeding during the close-up period for cows in the Overfed group. The reverse was evident for the Controlfed group with the greatest PMN at the highest intakes during the close-up period. The postpartum anoestrous interval (PPAI) was shorter in cows from the Overfed group when moderately fed (Feed90) during the close-up period; in comparison, the PPAI was shorter in the Controlfed group, when those cows were overfed in the close-up period (Feed120). The cows in the Overfed treatment had greater conception and pregnancy rates if cows had moderate dry matter intakes (Feed90) during the close-up period; these reproductive variables were less under excessive feed intakes (Feed120); yet, close-up dry matter intake had little effect on conception and pregnancy rates for the Controlfed group.

**Conclusions:**

The far-off feeding strategies increased early reproductive outcomes at 3 weeks of mating. Additionally, the interaction between far-off and close-up feeding strategies resulted in high six-week pregnancy rate with a slight restriction during the close-up period but only in the far-off Overfed group. Thus, our hypothesis is supported under these conditions.

## Background

In seasonal pasture-based dairy production systems, cows must recover from calving and become pregnant within two to 3 months. To achieve this, the uterus must involute, and ovulation must occur, then continue at regular intervals [1]. Nutritional or metabolic status post-calving contributes to these processes, particularly through management of pre- and post-calving body condition score (BCS) [2, 3]. Low BCS pre-calving is associated with endometritis (i.e., elevated polymorphonucleated (PMN) cells in the uterine lumen [4, 5]), an extended anovulatory period after calving [6, 7], and reduced pregnancy rates [8].

In grazing systems, cows are thinner at the end of lactation than housed cows fed a total mixed ration [8]. Therefore, cows need a greater level of feeding in late lactation and the far-off dry period to ensure calving BCS targets are achieved. But, there is evidence that over-feeding during the far-off dry period results in metabolic profiles that would negatively affect reproduction [9, 10]. During the close-up dry period (or early transition period) over-feeding is also associated with metabolic distress. Small restrictions in energy intake before calving have been reported to improve early lactation metabolic profiles [11, 12] and could, potentially, reduce any negative effects of overfeeding in the far-off period.

Roche et al. [13] reported a small, but unexplained, effect of differentially managing cows during the far-off, non-lactating period on metabolic health. Specifically, reduced circulating concentrations of non-esterified fatty acids (NEFA) and β-hydroxybutyrate (BHB) were observed in cows overfed in the far-off non-lactating period despite being at a similar BCS at calving [13]. Additionally, those cows from the Overfed group had increased albumin to globulin ration compared to the Controlfed post-calving [13]. Although a slight restriction (10–25%) during the close-up non-lactating period had beneficial effects on metabolic health and inflammatory state, a restriction in energy intake of > 35% for 3 weeks before calving reduced milk production and increased the blood concentration of indicators of metabolic stress and inflammation post-calving [13]. Based on the metabolic changes discussed in Roche et al. [13], we hypothesized that the feed intake during the far-off non-lactating period would have little effect on reproductive performance in the next lactation; but, a slight to moderate restriction (10–25%) during the close-up non-lactating period could be beneficial to reproductive function, while a more restrictive feeding strategy (> 35%) would result in adverse effects on reproductive outcomes.

## Methods

### Animals and feeding treatments

**Fig. 1:**
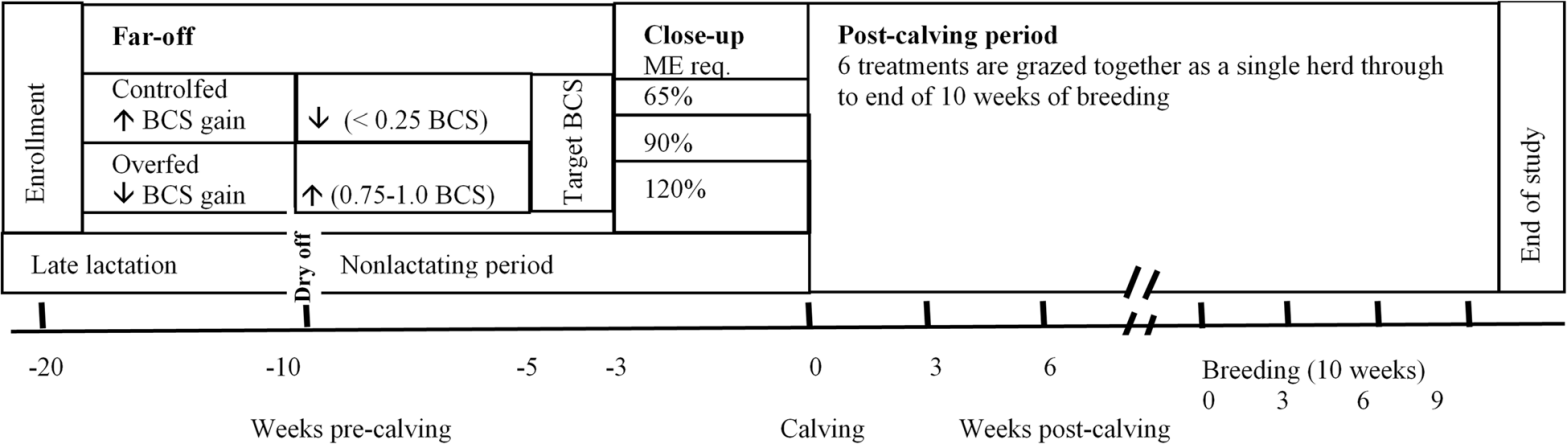
Schematic of the experimental periods. Far-off Controlfed group gained BCS during late lactation period, and during the nonlactating period this group was managed to gain < 0.25 BCS (Controlfed), the Overfed group gained little during the late lactation period and during the nonlactating period were managed to gain between 0.75 to 1.0 BCS (Overfed). Both groups achieved similar BCS approximately 5 weeks pre-calving. The close-up period from 3 weeks pre-calving where cows were managed to achieve 3 levels of ME intake (65% = Feed65, 90% = Feed90, and 120% = Feed120). Post-calving period where cows were managed in a single herd through to the end of the 10-week seasonal breeding period

We report a retrospective study evaluating reproductive performance of cows where the original study investigated the effect of far-off and close-up transition period feeding strategies on milk production and circulating indicators of energy balance and metabolic health [13]. This retrospective study is therefore exploratory as the main study was setup to detect differences in milk production, indicators of energy balance and metabolic health, and may therefore be underpowered for binary reproductive outcomes. The complete details of the experimental design are reported by Roche et al. [13]. To achieve this, a group of healthy 150 mid-lactation dairy cows of mixed age and breed (Holstein-Friesian, Jersey, Holstein-Friesian × Jersey) were allocated randomly to one of two treatment groups (75 cows per group) 18–20 weeks before planned start of calving. Although cow allocation to treatment was random, groups were assessed to ensure they were balanced for age, breed, BCS at the time of enrollment and expected calving date. The study was designed to have two feeding levels during the late lactation period and nonlactating period to generate 2 BCS treatment groups (Far-off; Controlfed, Overfed). During the late lactation, the Controlfed consumed 17.7 kg of DM/d (their period of accelerated gain to achieve BCS 4.75 on a 10-point scale, where 1 is emaciated and 10 is obese [14];) and during the far-off nonlactating period this group was managed to gain very little BCS (< 0.25 BCS) and consumed 8.4 kg of DM/d. Whereas, the Overfed group received 10.3 kg of DM/d during the late lactation period to achieve a BCS of approximately 4.25 and were to gain between 0.75 to 1.0 BCS after dry-off and 14.1 kg of DM/d during the nonlactating period (their period of BCS gain). The groups reaching similar BCS 4 to 5 weeks pre-calving. The nonlactating period started at 79 ± 9.5 d (11–12 weeks) before calving when cows were dried off. From 19 ± 5.4 d pre-calving (3 weeks; close-up period), cows within each far-off feeding group were randomly assigned to one of three levels of feed intake in a 2 × 3 factorial arrangement of treatments (Fig. 1). For the close-up period, feed allowances were managed to achieve three levels of ME intake up until calving (65%, 90%, and 120% of estimated ME requirements [15]: Feed65, Feed90, and Feed120, respectively). On average this equated to an average intake of 6.4, 8.7, and 10.6 kg DM/d (ME intake of 80±7.6, 109±14.0, and 132±16.5 MJ ME/d) [13]. The groups predominantly grazed pasture, during the far-off period cows were fed pasture, maize silage and palm kernel extract, close-up (pre-calving) cows received pasture and pasture silage, and pasture and pasture silage post-calving. Detailed these feeds and their composition, as well as the group BCS and body weights are reported by Roche et al. [13].

### Uterine health

Uterine samples were collected for endometrial cytology at 14 ± 0.1 d and 35 ± 0.1 d postpartum (mean ± SEM) as described by Meier et al. [16]. Briefly, the vulva of the cow was cleaned with a paper towel and a double-guarded, modified artificial insemination pipette was passed through the cervix and into the uterus. A stylet, with a cytology brush attached (Pap endocervical sample brush; EBOS Group Ltd., Christchurch, New Zealand), was used to collect a sample from the uterine wall. The stylet was retracted into the artificial insemination sheath and all sampling equipment removed from the cow. The brush was rolled onto a microscope slide and its contents air-dried. The dry slides were stained using Diff-Quick (Dade Behring Inc., Newark, DE). A single veterinary pathologist from Gribbles Veterinary Pathology Laboratory determined the proportion of PMN on the 318 slides taken. Areas of each slide that contained small clusters of epithelial cells (5 to 20 per cluster) were preferentially selected and all identifiable nucleated cells counted (485 ± 4.2 cells per slide; mean ± SEM). The PMN threshold for classifying cows with subclinical endometritis was ≥14% at d 14 and ≥ 7% at the second cytological d 35 [17].

On the completion of endometrial sampling, vaginal content was sampled using a Metricheck device (Simcro Tech Ltd., Hamilton, New Zealand; http://www.simcro.com/Product_Range/Specialised_Items/index.html); this device consists of a 40-mm diameter hemisphere of silicon attached to a 500-mm-long stainless steel rod. The vaginal content was scored from a range of 1 to 5, where 1 being clear mucus, and 5 being purulent pus [18];). We used the score of 0 where no sample was obtained. Cows were classified as having endometritis when the vaginal mucus was scored as > 1 and defined as having normal vaginal discharge when scored ≤1 [18].

### Milk progesterone concentrations & postpartum anoestrous interval (PPAI)

Progesterone was measured in milk twice weekly (a.m. milking on each Tuesday and Friday) from 14 d in milk through to Sep 30. Progesterone concentrations were determined using a commercial radioimmunoassay kit (Progesterone Coat-A-Count; Siemens, Los Angeles, CA). The average intra-assay coefficients of variation were 8.7% and 20.9% for the high and low controls, respectively and the average inter-assay coefficients of variation were 10.5% and 3.5% for the high and low controls (*n* = 11 assays), respectively. The minimum detectable concentration of progesterone was 0.07 ng/mL. The PPAI was defined as the interval from calving to the first of two consecutive samples with progesterone concentration ≥ 1.95 ng/mL [19]. Milk progesterone analyses were halted as cows reached the threshold above.

### Mating, synchronization, and pregnancy diagnosis

Reproductive management was undertaken as per farm policy. As the grazing system was seasonal, mating management commenced on a set calendar date (planned start of mating); the duration of artificial mating period was six weeks, with natural mating using bulls for a further 30 d. Thirteen cows at three time points (*n* = 2 Sep 30, *n* = 8 Oct 7, and *n* = 3 Oct 15) received a synchronization programme, as part of the farm management practice where once the cows completed the milk progesterone sampling period. The programme included an controlled internal drug release device into the vagina (CIDR®, 1.38 g progesterone; Zoetis NZ Ltd., Auckland, New Zealand) and an i.m. injection of 100 μg gonadorelin acetate (2 mL; Gonasyn, Agrihealth NZ Ltd., Auckland, New Zealand) at d 0. The progesterone insert was removed at d 7, followed by an i.m. injection of 500 μg cloprostenol Na-salt (2 mL, Cyclase, Agrihealth NZ Ltd) and 400 IU equine chorionic gonadotrophin i.m. (2 mL Novormon, Agrihealth NZ Ltd). Cows were mated on observed heat, and those cows that were unmated at d 9, received an i.m. injection of 100 μg gonadorelin acetate (2.0 mL; Gonasyn, Agrihealth NZ Ltd). Eleven cows (*n* = 3 Overfed-Feed120; *n* = 1 Overfed-Feed90, *n* = 2 Overfed-Feed65, *n* = 2 Controlfed-Feed120, *n* = 3 Controlfed-Feed65) identified with endometritis/metritis by farm staff (evaluated using Metricheck and via temperature where relevant [5];) prior to the start of mating received an intrauterine infusion of cephapirin (benzathine salt, 500 mg, Bomacure, Bayer NZ Limited, Auckland, New Zealand).

Pregnancy diagnoses, including fetal aging, were performed using ultrasonography with a 4.5 MHz - 8.5 MHz probe (Easi-Scan, BCF Technology Ltd., Scotland, UK). Fetal aging was undertaken 84 to 85 d after the planned start of mating, with cows not detected pregnant at that time allocated to an additional pregnancy diagnosis 52 d after the end of mating. Conception rate to any insemination was confirmed by foetal aging. Six cows conceived to an earlier mating date than the last recorded mating, with the earlier mating date used as the correct conception date.

### Statistical analysis

Statistical analyses were performed using SAS 9.3 (SAS Institute Inc., Cary, NC) and significance was declared if *P* ≤ 0.05, with a trend reported if *P* ≤ 0.10. Data are presented as means and the standard error of the mean (SEM), unless otherwise stated.

Metricheck score and PMN percent were subjected to repeated measures analysis of variance using mixed models (Proc Mixed, SAS/STAT 12.1). The models included far-off feeding strategy (Overfed or Controlfed), close-up feeding level strategy (Feed65, Feed90, or Feed120), week (two or five), and their interactions as fixed effects, and cow as a random effect. Breed (Holstein-Friesian or Holstein-Friesian-Jersey crossbred), and age group (4, 5, 6, ≥7 years) were used as blocking factors, and season-day (difference between actual calving date and the first calving cow in the group) as a covariate. Log_10_ and Probit transformations were used to achieve homogeneity of variance. The percentage of cows identified with purulent vaginal discharge (PVD) or subclinical endometritis were analysed using generalized linear mixed model (PROC GLIMMIX, SAS/STAT 12.1) to estimate the effects of far-off feeding level strategy (Overfed or Controlfed), close-up feeding level strategy (Feed65, Feed90, or Feed120), and their interaction after correcting for breed, age group, and season-day.

For cows that had ovulated, the interval from calving to ovulation, was subjected to two-way ANOVA, with far-off feeding level strategy (Overfed or Controlfed), close-up feeding level strategy (Feed65, Feed90, or Feed120), and their interaction included as fixed effects, breed and age group as blocking factors, and season-day (difference between actual calving data and first calving cow in the group) as a covariate. Data were log_10_ transformed before analysis to achieve homogeneity of variance. For cows that had ovulated, survival analysis (Proc Lifetest, SAS/STAT 12.1) was used to evaluate the effect of far-off feeding level strategy (Overfed or Controlfed) or close-up feeding level strategy (Feed65, Feed90, or Feed120) on time to ovulation. Season-day, breed, and age group were used as covariates.

Reproductive outcomes were evaluated by the parameters; 3-week submission rate, conception rate (CR) to first mating, conception rate during the first 3- and 6-week of mating (where CR is a measure of a cow’s fertility at service and is the number of pregnant cows divided by the total number of inseminations for the conditions outlined (i.e.. first mating or over the 3-week of mating), and 3-, 6- and final pregnancy rates (PR; where PR is the pregnant cows divided by the number of cows available). These were analysed using generalized linear mixed model (PROC GLIMMIX, SAS/STAT 12.1) to estimate the effects of far-off feeding level strategy (Overfed or Controlfed), close-up feeding level strategy (Feed65, Feed90, or Feed120), and their interaction after correcting for breed, age group, oestrus synchronization (None, Round 1, 2, or 3), Bomacure use, and season-day. For cows that had conceived, survival analysis (Proc Lifetest, SAS/STAT 12.1) was used to evaluate the effect of far-off feeding level strategy (Overfed or Controlfed) or close-up feeding level strategy (Feed65, Feed90, or Feed120) on time to ovulation. Season-day, breed, and age group were used as covariates.

## Results

### Uterine health

There was no effect of far-off treatment on the average Metricheck score (*P* = 0.95), and only a week tendency for an effect of the close-up treatment (*P* = 0.16) and not interaction (*P* = 0.35). Average Metricheck score was greater in the Feed65 (1.30 ± 0.07), followed by Feed120 (1.26 ± 0.08), and then Feed90 (1.12 ± 0.07), respectively.

There was an interaction between far-off and close-up treatment with the average PMN% (*P =* 0.025), with the far-off and close-up effect not significant (*P >* 0.36). The interaction was such that the lowest PMN% were recorded in the Overfed-Feed120 (18.69% ± 0.03%), then Controlfed-Feed90 (20.79% ± 0.03%), Controlfed-Feed90 (21.1% ± 0.03%), Controlfed-Feed65 (22.03% ± 0.03%), Controlfed-Feed120 (27.13% ± 0.03%), and Overlfed-Feed65 (30.56% ± 0.03%), respectively.

Both the Metricheck score and PMN% declined as the uterus recovered after calving (Metricheck 1.39 ± 0.07 at 2-week to 1.06 ± 0.03 at 5-week *(P <* 0.001); PMN% 37 ± 2.4 to 10% ± 1.0% (*P <* 0.001), respectively).

The far-off or close-up feeding strategy did not affect the proportion of cows with PVD (Metricheck score > 1 at 2 and 5 weeks postcalving), nor the proportion of cows with subclinical endometritis (subclinical endometritis; > 14% PMN at 14 d, or > 7% PMN at 35 d after calving). Two weeks after calving the groups ranged as follows; Overfed-Feed120 (7.8%), Controlfed-Feed90 (16.3%), Controlfed-Feed65 (19.4%), Controlfed-Feed120 (21.2%), Overlfed-Feed65 (28.5%) and Overfed-Feed120 (30.3%), respectively (Far-off *P =* 0.91, Close-up *P = 0*.21). At 5 weeks postcalving, proportions with PVD ranged from 0 to 7% across the groups. In increasing proportions of PVD at 5 weeks post-calving were; Overfed-Feed90 (0%), Overfed-Feed120 (0%), Controlfed-Feed90 (3.5%), Controlfed-Feed120 (4.1%), Controlfed-Feed65 (6.3%), and Controlfed-Feed65 (7.1%), respectively (Far-off *P =* 0.97, Close-up *P =* 0.99).

Subclinical endometritis did not differ with far-off nor close-up treatments at either 2 or 5 weeks postcalving, nor where there any interactions (*P* > 0.40 for all). At 2 weeks postcalving between 55% to 76% of cows had PMN > 14%, and at 5 weeks postcalving between 31% to 56% of cows had PMN > 7%. At 2 weeks post-calving the proportion with subclinical endometritis in increasing proportions were; Overfed-Feed120 (54.7%), Overfed-Feed90 (61.9%), Controlfed-Feed65 (66.1%), Controlfed-Feed100 (71.5%), Controlfed-Feed90 (75%) and Overfed-Feed65 (75.8%), respectively. At 5 weeks post-calving the proportions classified with subclinical endometritis declined to 31% to 57%. The groups ranked as follows; Controlfed-Feed90 (31.3%), Overfed-Feed120 (37.5%), Controlfed-Feed65 (40.5%), Overfed-Feed90 (47.5%), Controlfed-Feed120 (50.4%), and Overfed-Feed65 (56.5%), respectively.

### Postpartum anoestrous interval (PPAI)

**Fig. 2:**
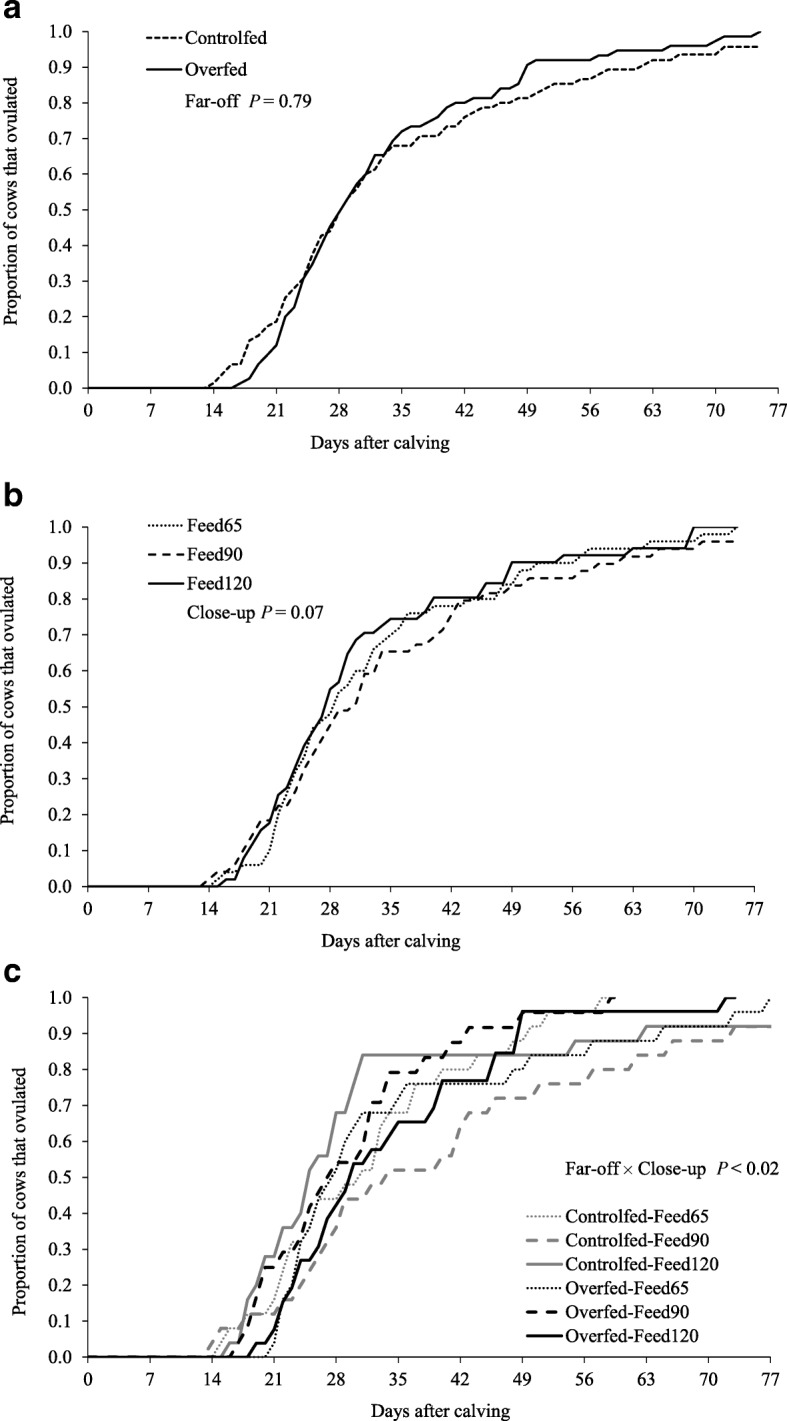
Effects of far-off and close-up transition feeding strategies on the cumulative proportion of cows ovulating post-calving. **a** far-off feeding strategies (Controlfed and Overfed) and **b**) of close-up intake (Feed65, Feed90 and Feed120) and, **c**) their interaction on the on the cumulative proportion of cows ovulating after calving. Details of the feeding strategies are outlined by Roche et al. [13]. Far-off *P* = 0.79; close-up *P* = 0.07; far-off × close-up *P <* 0.02

**Fig. 3:**
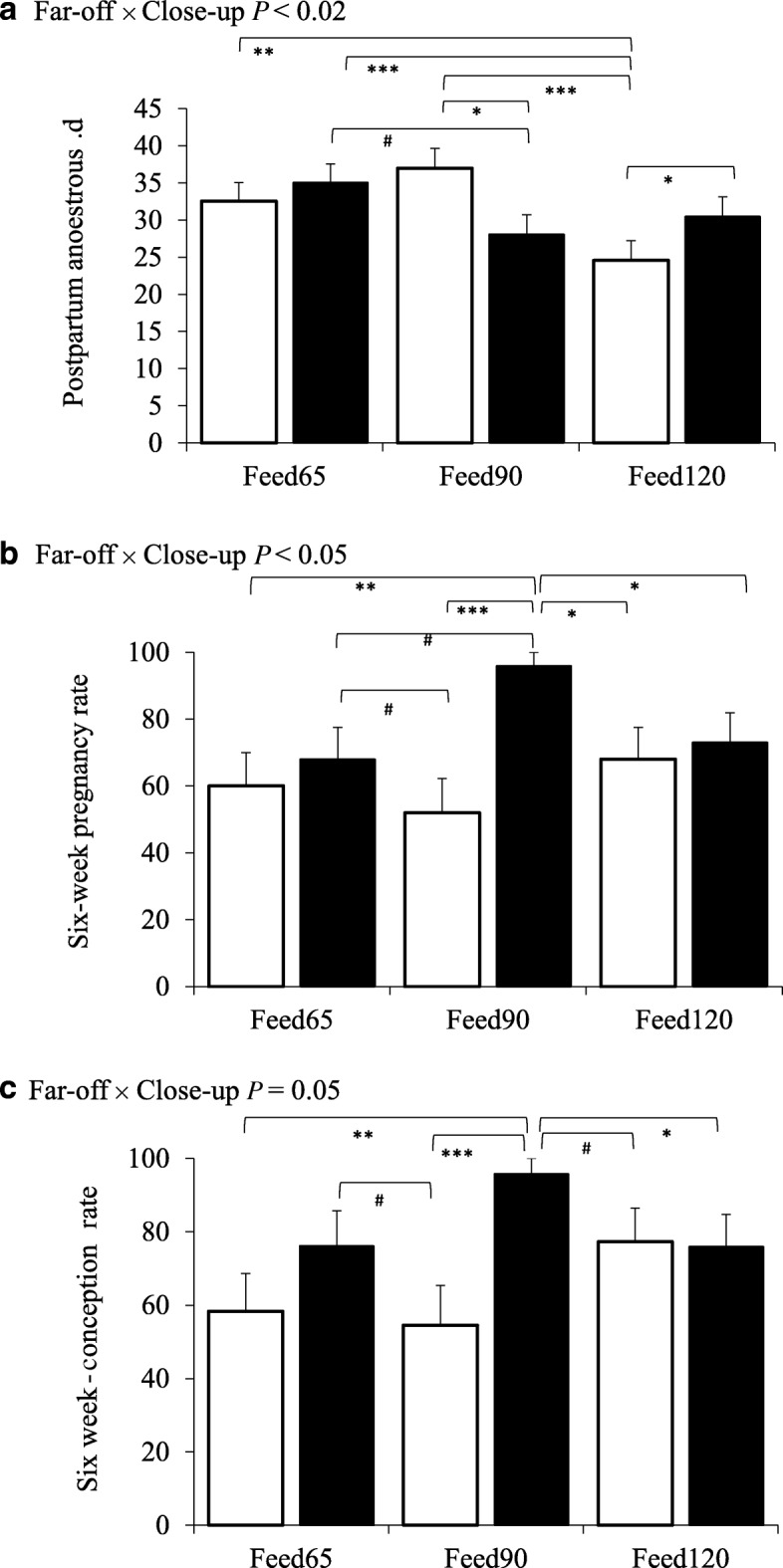
Interactions between feeding level during the far-off and close-up transition periods reproductive outcomes. Controlfed [white bar] and Overfed [black bar] depict the far-off treatments, and close-up feeding treatments are depicted as Feed65, Feed90, and Feed120. **a** postpartum anestrous (*P <* 0.02), **b** conception rate (*P =* 0.05), and **c** pregnancy rate after 6 weeks of mating (*P <* 0.05). Details of the feeding strategies see Roche et al. [13]. Significance *** *P* < 0.001, **P < 0.01*P < 0.05 ^#^*P <* 0.10 and *>* 0.05. Error bar represent standard error of the difference

Overall PPAI was 32 ± 1.1 d (range, 14 to 75 d). The Far-off feeding strategies did not affect the cumulative proportion of cows in oestrus with increased days in milk (Fig. 2); neither did they affect the average PPAI (31 d for the Controlfed and 31 d for Overfed treatments, respectively; SEM = 1.5 d; Table 1). In the Feed120 treatment there was a tendency for PPAI was 5 to 6 d less than the Feed65 and Feed90 treatments (*P* = 0.07; Table 1). The far-off and close-up feeding strategies interacted (*P <* 0.01), with a shorter PPAI in Overfed-Feed90 cows compared with Controlfed-Feed90 cows; in comparison, PPAI was shorter in the Controlfed-Feed120 group compared with Overfed-Feed120 cows (Fig. 2; Fig. 3a).

**Table 1:** Effects of far-off and close-up feeding strategies on reproduction performance in seasonal calving cows

Treatments	Far-off^a^			Close-up^b^				*P* values		
Groups	Controlfed	Overfed	SEM	Feed65	Feed90	Feed120	SEM	Far-off	Close-up	Interaction^c^
Postpartum anoestrus interval, d ^d^	31	31	1.6	34	33	28	1.9	0.80	0.07	< 0.02
3-week submission rate, %	91	93	3.4	90	94	92	4.3	0.99	0.99	0.75
Conception rate to first mating, %	44	61	5.8	46	57	55	7.1	< 0.05	0.75	0.64
Conception rate during first 3 weeks of mating, %	47	64	6.1	44	61	62	7.5	0.05	0.42	0.36
Conception rate during the first 6 weeks of mating, %	63	83	5.9	67	76	77	7.1	< 0.02	0.51	0.05
3-week pregnancy rate, %	43	60	5.8	40	57	57	7.1	0.06	0.37	0.33
6-week pregnancy rate, %	60	79	5.7	64	74	71	6.9	0.02	0.46	< 0.05
Final pregnancy rate, %^e^	79	91	4.8	82	90	82	5.5	0.10	0.47	0.69

^a^Far-off Controlfed (Controlfed) were fed to gain BCS during late lactation and maintain BCS during the far-off non-lactating period. Far-off Overfed (Overfed) were fed to maintain BCS during the late lactation and gain BCS during the far-off non-lactating period [13]

^b^Close-up treatments were managed to achieve daily ME intakes equivalent to 65%, 90% and 120% of their requirements (Feed65, Feed90, Feed120) during the 3 weeks pre-calving [13, 16]

^c^Interactions are detailed in Fig. 3a-c

^d^Interval from calving to d 1 of the two consecutive d that progesterone was > 1.95 ng/mL

^e^Mating period was 70 d (artificial insemination 39 d, 31 d natural bull mating)

### Reproductive performance (submission, conception, and pregnancy rates)

Over 90% of cows were submitted for mating in the first 3-week of mating and submission rate was not affected by either the far-off or the close-up feeding strategies (Table 1). The Overfed group during the far-off dry period improved CR to first mating (*P ≤* 0.05), and 3-week CR (*P =* 0.05) and PR (*P =* 0.06) by 17% when compared with the Controlfed group (Table 1). By the end of the mating period, there was a tendency for high pregnancy rates in the far-off Overfed treatment compared with the Controlfed group (final PR 91% for Overfed and 79% for the Controlfed groups, respectively; *P =* 0.10).

There was an interaction (*P =* 0.05; Table 1) between far-off and close-up feeding strategy on CR during the first 6 weeks of the mating period (Fig. 3bc). The interaction for 6-week CR was such that the lowest CR were achieved by the Controlfed-Feed65 and Controlfed-Feed90, with an increase in the Controlfed-Feed120 (Controlfed-Feed65, 58%, Controlfed-Feed90, 55% and Controlfed-Feed120, 77%, respectively; Fig. 3b). The greatest CR occurred in the Overfed-Feed90 group, followed by Overfed-Feed65 and 120 (CR in increasing order being; Overfed-Feed65, 76%, Overfed-Feed120, 76% and Overfed-Feed90, 96%, respectively; Fig. 3b).

**Fig. 4:**
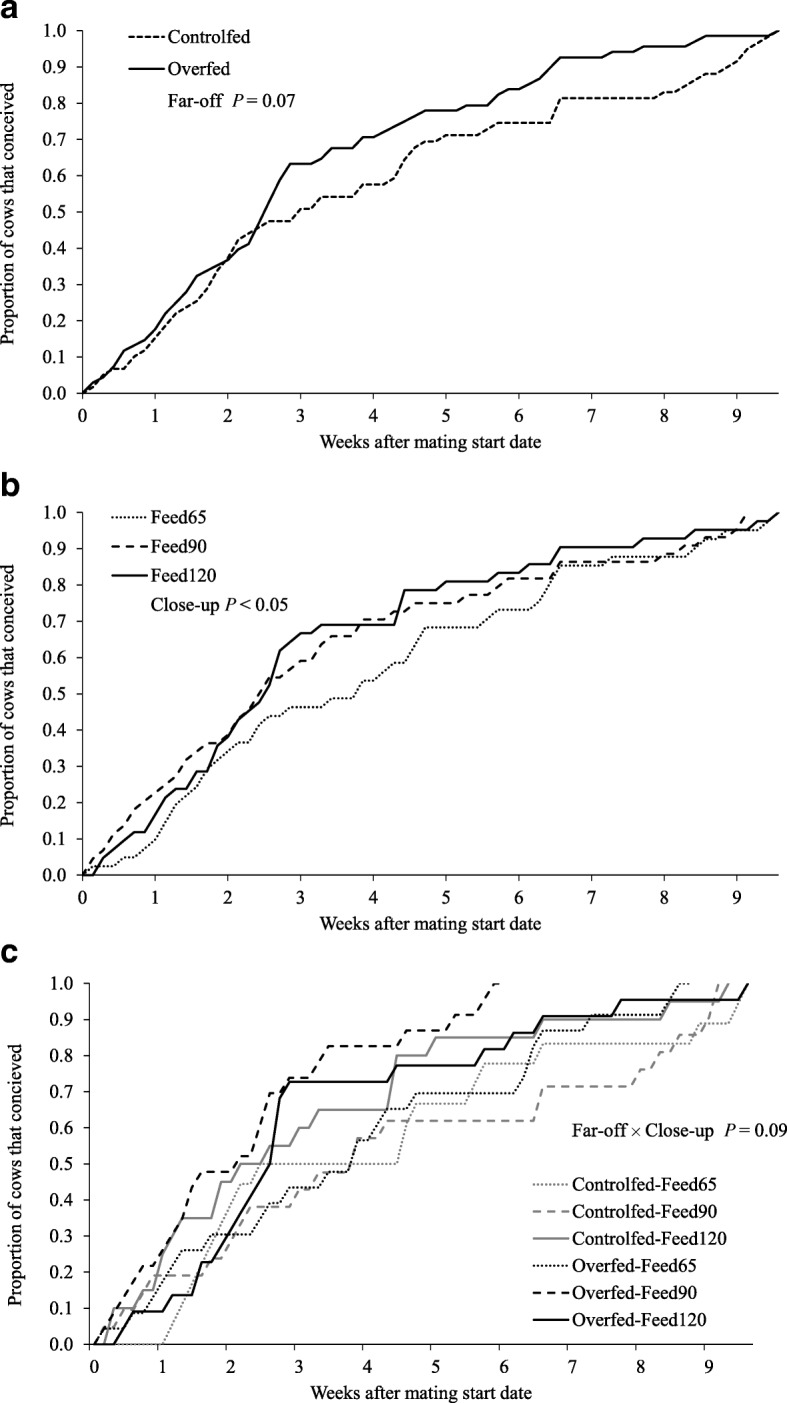
Effects of far off and close-up feeding strategies on the cumulative proportions of cows that conceived. **a** far-off feeding strategies (Controlfed and Overfed); and **b** of close-up intake (Feed65, Feed90 and Feed120), and, **c** their interaction on the cumulative proportion of cows that conceived for mating period. Details of the feeding strategies are outlined by Roche et al. [13]. Far-off *P* = 0.07; close-up *P* < 0.05; far-off × close-up *P* = 0.09

The interaction for 6-week PR was similar (*P <* 0.05; Table 1) for 6-week PR improved in the Overfed-Feed90 group compared with the Overfed-Feed65 and Overfed-Feed120 groups (Overfed-Feed65, 68%, Overfed-Feed90, 96% and Overfed-Feed120, 73% pregnant after 6 weeks of mating, respectively; Fig. 3c, and Fig. 4). Pregnancy outcomes were also greater in the Overfed–Feed120 treatment group compared with any of the close-up feeding levels (Controlfed-Feed120; 68% 6-week PR compared with 60% and 52% for the Controlfed-Feed65 and Controlfed-Feed90, respectively).

The interaction between far-off and close-up treatments on the interval from mating to conception was not significant (*P =* 0.166). The interval from mating to conception ranged from 14 d to 32 d. In increasing order from 14 d, to 32 d were Overfed-Feed90 (14 d), Overfed-Feed65 (18 d), Controlfed-Feed120 (27 d), Overfed-Feed120 (29 d), Controlfed-Feed90 (30 d) and Controlfed-Feed65 (32 d), respectively (Fig. 4).

## Discussion

We hypothesized that the level of feed intake during the far-off non-lactating period would not affect reproductive performance in the next mating. The results of this study support this hypothesis for uterine health outcomes but demonstrate that feed intake during the far-off non-lactating period does affect reproductive performance early in the mating period. Furthermore, we hypothesized that close-up feeding strategies would affect reproductive outcomes. Although true, the effect was subject to an interaction with far-off feeding strategies. This interaction was significant for the duration of PPAI and the conception and pregnancy rates after 6 weeks of mating; the results highlight a complexity of pre-calving feed intakes on reproductive outcomes. The results support an alternative hypothesis that both far-off and close-up feeding strategies affect reproductive outcomes, and that the shortest PPAI and highest pregnancy outcomes arise from high feed intakes during the far-off non-lactating period in combination with a slight restriction during the close-up period (Overfed × Feed90).

The results demonstrate that the far-off non-lactating feed intakes affected reproductive outcomes during the first 3 weeks of mating. Specifically, the cows overfed in the far-off, non-lactating period had > 10% improvement in CR and PR when compared with the Controlfed cows. We did not investigate the mechanisms behind this significant increase in conception and pregnancy rates and, so, cannot say, with any certainty, why these effects occurred. However, the level of feeding during the far-off dry period has been reported to modify metabolic status early post-calving in ways that could be expected to affect reproductive function: cows overfed in the far-off period were reported to have a less severe negative energy balance after calving, they lost less BCS, and they had reduced blood NEFA and BHB [13]; consistent with this metabolic profile, their adipose tissue was, also, more primed to retain rather than release fatty acids [20]. A more severe negative energy balance after calving, as well as elevated NEFA and BHB, have been reported to reduce CR and PR [21, 22].

Although the direction of change in reproductive outcomes is consistent with the metabolic changes reported by Roche et al. [13]), the treatment effects on BCS, NEFA, and BHB would not be expected to have such a large effect on reproduction [8]. It is possible, therefore, that the effects reported here are a result of other long-term effect of nutrition on reproductive function as suggested by Britt [23], wherein the nutritional status 80–100 d prior to measurements affects oocyte quality. The BCS change profile reported by Roche et al. [13] confirmed that cows overfed during the far-off non-lactating period were in a considerably more positive nutritional state which supported a faster rate of BCS gain compared with control fed cows. It is plausible, therefore, that this nutritional state positively affected the development of the primordial and primary follicles [23, 24] and, in this way, improved reproductive outcomes in cows overfed during the far-off non lactating period, but calving at optimal BCS. The negative effects of severe feed restriction (Feed65) on reproductive outcomes (such as extended PPAI) are supported by previous studies [6, 7, 12]. Also, the metabolic profile of the restricted group (Feed65) is indicative of severe negative energy balance post calving [13], which negatively affects reproductive success and should be avoided.

The low level of significance was not unexpected due to the size of the study, which as small and underpowered to generate robust results related to binary reproductive outcomes. However, this study still provides relevant information for future work. The level of feeding during both the far-off and close-up non-lactating periods affected the duration of the PPAI, and CR and PR after six-week of mating. In general, the interaction was such that cows overfed during the far-off dry period, followed by a slight restriction during the close-up period (Overfed-Feed90 cows), had a shorter PPAI and increased CR and PR after six-week of mating. In contrast, however, the far-off control-fed group demonstrated the shortest PPAI when they received the highest close-up period feed allowance (Feed120). The results reported here are limited by the scale of the study. Due to the scale of the current study, we propose that further studies be considered; first, to confirm the effects of far-off and close-up feeding strategies on reproductive outcomes at a larger scale, and second; to target the mechanisms that contribute to the complex interactions between the far-off and close-up feeding strategies and reproductive outcomes with a focus on providing effective solutions that can be adopted by farmers.

## Conclusions

Our results indicate that far-off and close-up feeding levels interact to produce the best reproductive outcomes (cows pregnant by 6 weeks of mating). The combination of overfeeding during the far-off period and a slight feed restriction during the close-up dry period results in cows with a shortened PPAI and those with greatest chance of pregnancy. Also, cows offered reduced feed intakes during the far-off period, and greater feed intakes during the close-up period had better reproductive outcomes with reduced the PPAI and improved pregnancy rates. Detrimental effects on reproductive outcomes were evident when cows were severely restricted during the close-up dry period or overfed during both far-off and close-up periods.

## Data Availability

The datasets used and/or analysed during the current study are available from the corresponding author on reasonable request.

## Abbreviations

BCS: Body condition score
BHB: β-hydroxybutyrate
Controlfed: Control fed during the far-off transition period
CR: Conception rate
DMI: Dry mater intake
Feed120: Feeding level during the close-up transition period (1 month pre-calving) with intakes of 120% of metabolizable energy relative to pre-calving requirements
Feed65: Feeding level during the close-up transition period (1 month pre-calving) with intakes of 65% of metabolizable energy relative to pre-calving requirements
Feed90: Feeding level during the close-up transition period (1 month pre-calving) with intakes of 90% of metabolizable energy relative to pre-calving requirements
ME: Metabolizable energy
NEFA: Non-esterified fatty acids
Overfed: Overfed fed during the far-off transition period
PMN: Polymorphonucleated cells
PPAI: Postpartum anoestrus interval
PR: Pregnancy rate
SED: Standard error of the difference
SEM: Standard error of the mean

## References

[1] Verkerk G (2003). Pasture-based dairying: challenges and rewards for New Zealand producers. Theriogenology.

[2] Buckley F, O'Sullivan K, Mee JF, Evans RD, Dillon P (2003). Relationships among milk yield, body condition, cow weight, and reproduction in spring-calved Holstein-Friesians. J Dairy Sci.

[3] Roche JR, Friggens NC, Kay JK, Fisher MW, Stafford KJ, Berry DP (2009). Invited review: body condition score and its association with dairy cow productivity, health, and welfare. J Dairy Sci.

[4] Roche JR, Macdonald KA, Schutz KE, Matthews LR, Verkerk GA, Meier S (2013). Calving body condition score affects indicators of health in grazing dairy cows. J Dairy Sci.

[5] McDougall S, Hussein H, Aberdein D, Buckle K, Roche J, Burke C (2011). Relationships between cytology, bacteriology and vaginal discharge scores and reproductive performance in dairy cattle. Theriogenology.

[6] Burke CR, Roche JR (2007). Effects of pasture feeding during the periparturient period on postpartum anovulation in grazed dairy cows. J Dairy Sci.

[7] Rhodes FM, McDougall S, Burke CR, Verkerk GA, Macmillan KL (2003). Invited review: treatment of cows with an extended postpartum anestrous interval. J Dairy Sci.

[8] Roche JR, Macdonald KA, Burke CR, Lee JM, Berry DP (2007). Associations among body condition score, body weight, and reproductive performance in seasonal-calving dairy cattle. J Dairy Sci.

[9] Cardoso FC, LeBlanc SJ, Murphy MR, Drackley JK (2013). Prepartum nutritional strategy affects reproductive performance in dairy cows. J Dairy Sci.

[10] Dann HM, Litherland NB, Underwood JP, Bionaz M, D'Angelo A, McFadden JW (2006). Diets during far-off and close-up dry periods affect periparturient metabolism and lactation in multiparous cows. J Dairy Sci.

[11] Loor JJ, Dann HM, Guretzky NA, Everts RE, Oliveira R, Green CA (2006). Plane of nutrition prepartum alters hepatic gene expression and function in dairy cows as assessed by longitudinal transcript and metabolic profiling. Physiol Genomics.

[12] Roche JR, Meier S, Heiser A, Mitchell MD, Walker CG, Crookenden MA (2015). Effects of precalving body condition score and prepartum feeding level on production, reproduction, and health parameters in pasture-based transition dairy cows. J Dairy Sci.

[13] Roche JR, Heiser A, Mitchell MD, Crookenden MA, Walker CG, Kay JK (2017). Strategies to gain body condition score in pasture-based dairy cows during late lactation and the far-off non-lactating period and their interaction with close-up dry matter intake. J Dairy Sci.

[14] Roche JR, Dillon PG, Stockdale CR, Baumgard LH, VanBaale MJ (2004). Relationships among international body condition scoring systems. J Dairy Sci.

[15] Roche JR, Kolver ES, Kay JK (2005). Influence of precalving feed allowance on periparturient metabolic and hormonal responses and milk production in grazing dairy cows. J Dairy Sci.

[16] Meier S, Priest NV, Burke CR, Kay JK, McDougall S, Mitchell MD (2014). Treatment with a nonsteroidal antiinflammatory drug after calving did not improve milk production, health, or reproduction parameters in pasture-grazed dairy cows. J Dairy Sci.

[17] Priest NV, McDougall S, Burke CR, Roche JR, Mitchell M, McLeod KL (2013). The responsiveness of subclinical endometritis to a nonsteroidal antiinflammatory drug in pasture-grazed dairy cows. J Dairy Sci.

[18] McDougall S, Macaulay R, Compton C (2007). Association between endometritis diagnosis using a novel intravaginal device and reproductive performance in dairy cattle. Anim Reprod Sci.

[19] Xu ZZ, Verkerk GA, Mee JF, Morgan SR, Clark BA, Burke CR (2000). Progesterone and follicular changes in postpartum noncyclic dairy cows after treatment with progesterone and estradiol or with progesterone, GnRH, PGF2α, and estradiol. Theriogenology.

[20] Vailati Riboni M, Farina G, Batistel F, Heiser A, Mitchell MD, Crookenden MA (2017). Far-off and close-up dry matter intake modulate the adipose tissue immunometabolic adaptation to lactation in pasture-based transition dairy cows. J Dairy Sci.

[21] Walsh RB, Walton JS, Kelton DF, LeBlanc SJ, Leslie KE, Duffield TF (2007). The effect of subclinical ketosis in early lactation on reproductive performance of postpartum dairy cows. J Dairy Sci.

[22] Umaña Sedó S, Rosa D, Mattioli G, Luzbel de la Sota R, Giuliodori MJ (2018). Associations of subclinical hypocalcemia with fertility in a herd of grazing dairy cows. J Dairy Sci.

[23] Britt JH (1992). Impacts of early postpartum metabolism on follicular development and fertility. Annu Conv Am Assoc Bovine Pract.

[24] Britt JH (1995). Effect of short- and long-term changes in energy balance on reproduction. Mid-South Ruminant Nutrition Conference.

[25] Ministry for Primary Industries (1999). New Zealand Animal Welfare Act: Use of animals in research, testing and teaching (Part 6).

